# Supplementary material for: Mobilization practices in the ICU: A nationwide 1-day point- prevalence study in Brazil
Source: PLoS One. 2020 Apr 2;15(4):e0230971. doi: 10.1371/journal.pone.0230971 (PMC7117707; doi:10.1371/journal.pone.0230971)
Supplement: S1 Text — (DOCX) [file pone.0230971.s001.docx]

**Mobilization practices in the ICU: A nationwide 1-day point- prevalence study in Brazil**

**Supporting Information**

### Karina T. Timenetsky; Ary Serpa Neto; Murillo S. C. Assunção, Leandro Taniguchi, Raquel A. C. Eid, Thiago D. Corrêa, for the e-MOTION Study group investigators.

**Study protocol**

**Objective:** To evaluate the prevalence of mobilization activities of critically ill patients in Brazilian ICUs through a nationwide one-day point prevalence study.

**Methods**

**Design and setting**

A 1-day, prospective, multicenter point prevalence study with a 24-hour follow-up period of mobilization activities of critically ill patients in Brazilian ICUs. The study was approved by the Hospital Israelita Albert Einstein’s Ethics Committee (CAAE: 43545015.3.1001.0071), and each site obtained ethics approval for the study. Informed consent was obtained for all patients as requested by the ethics committee.

**Participants selection**

Methods for recruitment of participating institutions included emailing members of the Brazilian Association of Intensive Care (Associação Brasileira de Medicina Intensiva, AMIB), announcements at national meetings and symposium, and emailing contacts and collaborators of each writing committee member. Adult patients (≥ 18 years old) were eligible for inclusion if they were expected to stay at ICU for at least 24 hours. Exclusion criteria were patients with terminal disease or pregnancy.

**Data collection and study variables**

A clinician not involved in the study randomly chose which weekday the study would take place by selecting one of five sealed opaque envelopes. Weekend days were excluded from the selection due to reduced staff and anticipated lower mobilization activities. All responsible researchers for each participating center were informed by email in the morning of the selected day in order to perform data collection for the 24-hour follow up.

Study data were collected and managed using Research Electronic Data Capture (REDCap) hosted at Hospital Israelita Albert Einstein [19]. Principal investigators of each participating ICU completed an online survey about the hospital and ICU characteristics, including type of hospital (public, private, and university), type of ICU (medical, surgical, mixed), number of ICUs beds, number of physiotherapists during a 6 hour shift, physiotherapist to patient ratio and nurse to patient ratio during a 6 hour shift, professional responsible for initiating patients’ mobilization (physician, nurse, physiotherapist) and presence of institutional early mobility, sedation and delirium protocols.

Collected variables included demographics, comorbidities, ICU admission diagnosis, Sequential Organ Failure Assessment (SOFA) score [20], supportive therapy (need for vasopressors, invasive mechanical ventilation and noninvasive mechanical ventilation) during index ICU stay, type of ventilatory support, use of sedation (if receiving any type of sedation) and mobilization activities. The following patients’ variables were related to the study day: SOFA, supportive therapy, type of ventilatory support, sedation practices and mobilization activities.

The study’s caser report form (CRF) are described in Appendix 1.

**Mobilization activities**

Mobilization activity was defined as any mobilization performed. Data on patients’ mobility were collected during a 24-hour period in a single day (June 29^th^ of 2017). Prevalence of mobilization activities, the highest level of mobilization performed during the study day (in bed or out of bed exercises), type of exercise performed (passive, assisted, active-assisted, active, and resisted exercises) and reasons for non performance of mobilization were collected. Contra indications for mobilization were considered as respiratory, cardiovascular, neurological or other considerations as described in the study published by Hodgson and colleagues [21].

**Statistical analysis**

Convenience sampling was used to include the patients in the study.

Categorical variables are presented as absolute and relative frequencies. Continuous variables are presented as median with interquartile ranges (IQR).

Logistic regression models were used to evaluate factors associated with mobilization activity and with out of bed exercises. Predictors (independent variables) included into the logistic regression models were SOFA score [20], the use of invasive and noninvasive mechanical ventilation, the use of vasoactive drugs, type of hospital, type of ICU, number of physiotherapists per 6-hour shift, number of patients per physiotherapist, and presence of institutional early mobility protocol. Multi-collinearity was checked for all variables. Results were presented as odds ratio (OR) along with 95% confidence interval (95%CI). Statistical tests were two-sided. A p<0.05 was considered statistically significant. All analyses were done in R (version 3.6.0).

**Appendix 1 – Online Survey (CRF)**

**ICU characteristics**

Dear colleague,

Thank you for participating in the study “Early mobilization in the ICU: a 1-day point-prevalence study in Brazil”**.**

We kindly ask you to fill out the flowing survey with your center information.

1. Center name:_________________________________
2. Type of hospital: ( ) Public ( ) Private ( ) University
3. Type of ICU: ( ) Medical ( ) Surgical ( ) Mixed
4. Country region: ( ) North

( ) Northeast

( ) Central-west

( ) Southeast

( ) South

1. Number of ICU beds:________
2. Number of physiotherapist per 6-hour shift:________
3. Physiotherapist to patient ratio during a 6 hour shift:________
4. Nurse to patient ratio during a 6 hour shift: ________
5. professional responsible to initiate patient’s mobilization:

( ) Physician ( ) Nurse ( ) Physiotherapist

1. Presence of institutional protocol for:

( ) early mobility ( ) sedation ( ) delirium

**Patients’ characteristics**

**Patient record ID number:____________ ( ) Inclusion date:___________**

**Age (years):______ ( ) ICU admission date:___________**

**Gender:** ( ) Male ( ) Female

**Reason for index ICU admission:** ( )Medical ( )Surgical ( )Mixed

**Reason for ICU admission:**

( ) Neurological

( ) Cardiologic

( ) Respiratory

( ) Gastric intestinal

( ) Metabolic

( ) Elective surgery

( ) Transplant

( ) Trauma

**SOFA score:_____**

**Hospital Category:** ( ) Public ( ) Private ( ) University

**Sedation:** ( ) Yes ( ) No

**Vasoactive drugs:** ( ) Yes ( ) No

**Ventilatory support:**

( ) Mechanical Ventilation ( ) Noninvasive Mechanical Ventilation

( ) No support

**Performing early mobilization**: ( ) Yes ( ) No

**Reason for no mobilization**:

( ) Contra indications (hemodynamic and respiratory instability, shock, medical restrictions)

( ) Absence of institutional early mobility protocol

( ) Unavailability of physiotherapist

( ) Absence of adequate equipment for mobility

( ) Physical space

( ) No recognition to perform early mobility by the ICU team

( ) Use of mechanical ventilation

( ) Use of hemodialysis

( ) Others

**Type of exercise performed:**

( ) Passive

( ) Assisted

( ) Active-assisted

( ) Active

( ) Resisted

**Safety events:**

( ) Respiratory distress

( ) Hemodynamic instability

( ) Accidental chest tube removal

( ) Accidental central venous catheter removal

( ) Accidental peripheral catheter removal

( ) Accidental chest drain removal

( ) Others

**Checklist**

**STROBE Statement**—Checklist of items that should be included in reports of ***cross-sectional studies***

|  | Item No | Recommendation | Page No |
| --- | --- | --- | --- |
| **Title and abstract** | 1 | (*a*) Indicate the study’s design with a commonly used term in the title or the abstract | 1 |
|  |  | (*b*) Provide in the abstract an informative and balanced summary of what was done and what was found | 2 |
| Introduction | | | |
| Background/rationale | 2 | Explain the scientific background and rationale for the investigation being reported | 4 |
| Objectives | 3 | State specific objectives, including any prespecified hypotheses | 4 e 5 |
| Methods | | | |
| Study design | 4 | Present key elements of study design early in the paper | 5 |
| Setting | 5 | Describe the setting, locations, and relevant dates, including periods of recruitment, exposure, follow-up, and data collection | 5,6,7 |
| Participants | 6 | (*a*) Give the eligibility criteria, and the sources and methods of selection of participants | 5 |
| Variables | 7 | Clearly define all outcomes, exposures, predictors, potential confounders, and effect modifiers. Give diagnostic criteria, if applicable | 6,7 |
| Data sources/ measurement | 8* | For each variable of interest, give sources of data and details of methods of assessment (measurement). Describe comparability of assessment methods if there is more than one group | 6,7 |
| 6,7Bias | 9 | Describe any efforts to address potential sources of bias | 7 |
| Study size | 10 | Explain how the study size was arrived at | 7 |
| Quantitative variables | 11 | Explain how quantitative variables were handled in the analyses. If applicable, describe which groupings were chosen and why | 7 |
| Statistical methods | 12 | (*a*) Describe all statistical methods, including those used to control for confounding | 7 |
|  |  | (*b*) Describe any methods used to examine subgroups and interactions | 7 |
|  |  | (*c*) Explain how missing data were addressed | 7 |
|  |  | (*d*) If applicable, describe analytical methods taking account of sampling strategy |  |
|  |  | (*e*) Describe any sensitivity analyses | 7 |
| Results | | | |
| Participants | 13* | (a) Report numbers of individuals at each stage of study—eg numbers potentially eligible, examined for eligibility, confirmed eligible, included in the study, completing follow-up, and analysed | 8 |
|  |  | (b) Give reasons for non-participation at each stage | 8,9 |
|  |  | (c) Consider use of a flow diagram | 8 |
| Descriptive data | 14* | (a) Give characteristics of study participants (eg demographic, clinical, social) and information on exposures and potential confounders | 9 |
|  |  | (b) Indicate number of participants with missing data for each variable of interest | 8 |
| Outcome data | 15* | Report numbers of outcome events or summary measures | 11,12,13 |
| Main results | 16 | (*a*) Give unadjusted estimates and, if applicable, confounder-adjusted estimates and their precision (eg, 95% confidence interval). Make clear which confounders were adjusted for and why they were included |  |
|  |  | (*b*) Report category boundaries when continuous variables were categorized | 12,13 |
|  |  | (*c*) If relevant, consider translating estimates of relative risk into absolute risk for a meaningful time period | ----- |
| Other analyses | 17 | Report other analyses done—eg analyses of subgroups and interactions, and sensitivity analyses | 11,12 |
| Discussion | | | |
| Key results | 18 | Summarise key results with reference to study objectives | 13,14 |
| Limitations | 19 | Discuss limitations of the study, taking into account sources of potential bias or imprecision. Discuss both direction and magnitude of any potential bias | 17 |
| Interpretation | 20 | Give a cautious overall interpretation of results considering objectives, limitations, multiplicity of analyses, results from similar studies, and other relevant evidence | 14 |
| Generalisability | 21 | Discuss the generalisability (external validity) of the study results | 17 |
| Other information | | | |
| Funding | 22 | Give the source of funding and the role of the funders for the present study and, if applicable, for the original study on which the present article is based | _____ |

*Give information separately for exposed and unexposed groups.

**Note:** An Explanation and Elaboration article discusses each checklist item and gives methodological background and published examples of transparent reporting. The STROBE checklist is best used in conjunction with this article (freely available on the Web sites of PLoS Medicine at http://www.plosmedicine.org/, Annals of Internal Medicine at http://www.annals.org/, and Epidemiology at http://www.epidem.com/). Information on the STROBE Initiative is available at www.strobe-statement.org.

**Table**

S1 Table: Multi-collinearity results for the multi regression analysis included variables in Table 3 and 4 of the manuscript.

|  | VIF Df | VIF^(1/(2*Df) |
| --- | --- | --- |
| Table 3 |  |  |
| SOFA | 2.427044  1 | 1.557897 |
| factor(vent_support) | 1.825397  2 | 1.162356 |
| factor(vasoactive) | 1.627499  1 | 1.275735 |
| number_physio | 2.438260  1 | 1.561493 |
| number_pat_physio | 2.266605  1 | 1.505525 |
| factor(protocol_insti) | 2.064531  1 | 1.436848 |
| factor(type_hospital) | 5.343080  2 | 1.520365​ |
| Table 4 |  |  |
| SOFA | 1.246340  1 | 1.116396 |
| factor(vent_support) | 1.106374  2 | 1.025594 |
| factor(vasoactive) | 1.193238  1 | 1.092354 |
| number_physio | 1.638603  1 | 1.280079 |
| number_pat_physio | 1.413337  1 | 1.188839 |
| factor(protocol_insti) | 1.515191  1 | 1.230931 |
| factor(type_hospital) | 1.701053  2 | 1.142035 |

SOFA (Sequential Organ Failure Assessment), VIF (variance inflation factor)

**S2 Table.** Multivariable logistic regression model of factors associated with mobilization activities in patients within 72 hours of ICU admission.

| **Variables** | **OR** | **95% CI** | **p value** |
| --- | --- | --- | --- |
| SOFA score | 0.83 | 0.67 - 1.02 | 0.072 |
| Ventilatory support |  |  |  |
| No support | 1.00 | (Reference) | --- |
| Invasive mechanical ventilation | 2.57 | 0.45 - 14.64 | 0.286 |
| Noninvasive ventilation | 7.61 | 1.29 – 44.62 | 0.024 |
| Use of vasoactive drugs | 1.19 | 0.29 – 4.93 | 0.803 |
| Type of hospital  Public  Private  University | 1.00  1.14  0.31 | (Reference)  0.15 - 8.89  0.04 - 2.27 | ---  0.889  0.251 |
| Number of physiotherapists per 6-hour shift | 0.58 | 0.33 - 1.02 | 0.059 |
| Number of patients per physiotherapist | 0.99 | 0.77 - 1.28 | 0.960 |
| Institutional early mobility protocol | 13.96 | 2.38 - 81.83 | 0.003 |

OR: Odds Ratio, 95% CI: 95% Confidence Interval, SOFA score: sequential organ failure assessment.

**S3 Table**. Multivariable logistic regression model of factors associated with out of bed exercise in patients within 72 hours of ICU admission.

| **Variables** | **OR** | **95% CI** | **p value** |
| --- | --- | --- | --- |
| SOFA score | 0.79 | 0.53 - 1.16 | 0.234 |
| Ventilatory support |  |  |  |
| No support | 1.00 | (Reference) | --- |
| Noninvasive ventilation | 0.15 | 0.02 – 1.15 | 0.068 |
| Invasive mechanical ventilation | 0.00 | 0.00 – -- | 0.980 |
| Use of vasoactive drugs  Type of hospital  Public  Private  University | 2.46  1.00  2.83  0.56 | 0.21 - 29.45  (Reference)  0.52 – 15.29  0.01 – 20.65 | 0.475  ---  0.225  0.755 |
| Number of physiotherapists per 6-hour shift | 1.87 | 1.14 – 3.08 | 0.012 |
| Number of patients per physiotherapist | 1.57 | 1.03 – 2.38 | 0.034 |
| Institutional early mobility protocol | 5.25 | 0.14 - 193.36 | 0.367 |

OR: Odds Ratio, 95% CI: 95% Confidence Interval, SOFA score: sequential organ failure assessment.

**S4 Table:** Multi-collinearity results for the multi regression analysis included variables in Table S2 and S3.

|  | VIF Df | VIF^(1/(2*Df) |
| --- | --- | --- |
| Table 3 |  |  |
| SOFA | 3.270502 1 | 1.808453 |
| factor(vent_support) | 3.092551 2 | 1.326109 |
| factor(vasoactive) | 1.773052 1 | 1.331560 |
| number_physio | 3.591615 1 | 1.895156 |
| number_pat_physio | 2.831585 1 | 1.682731 |
| factor(protocol_insti) | 3.109403 1 | 1.763350 |
| factor(type_hospital) | 9.303634 2 | 1.746478 |
| Table 4 |  |  |
| SOFA | 1.459635 1 | 1.208154 |
| factor(vent_support) | 1.373632 2 | 1.082599 |
| factor(vasoactive) | 1.456421 1 | 1.206823 |
| number_physio | 2.496919 1 | 1.580164 |
| number_pat_physio | 2.787808 1 | 1.669673 |
| factor(protocol_insti) | 2.650222 1 | 1.627950 |
| factor(type_hospital) | 3.386674 2 | 1.356574 |

SOFA (Sequential Organ Failure Assessment), VIF (variance inflation factor)
