# Supplementary material for: Mobilization practices in the ICU: A nationwide 1-day point- prevalence study in Brazil
Source: PLoS One. 2020 Apr 2;15(4):e0230971. doi: 10.1371/journal.pone.0230971 (PMC7117707; doi:10.1371/journal.pone.0230971)
Supplement: Attachment — Submitted filename: Response to Reviewers.docx [file pone.0230971.s002.docx]

São Paulo, January 24^th^, 2020.

Response to the Editor’s and the Reviewers’ comments

**PONE-D-19-29730
Mobilization practices in the ICU: A nationwide 1-day point- prevalence study in Brazil**

Dear Martina Crivellari,

Academic Editor of PLOS ONE

We would like to express our gratitude for giving us the opportunity to revise our manuscript. We believe the reviewers made insightful suggestions that have greatly helped address critical unresolved issues to improve the manuscript.

All the changes in the paper are highlighted in yellow in the revised version to indicate the revised portions of the manuscript. We made modifications in the manuscript according to the reviewers’ comments keeping it within the word and referenced limits imposed by the Journal. We have also revised the manuscript taking into consideration the request made by the Editorial Office. We expect our manuscript to be suitable now for publication in PLOS ONE.

I remain at your disposal for any clarification you might require.

Yours sincerely,

On behalf of all the authors,

Karina T. Timenetsky

**Reviewer #1:**

Thank you very much for your comments and suggestions.

1- Thank you for the opportunity to review this manuscript that deals with the important issue of mobilization of ICU patients. I do have some substantial comments that I believe require consideration and potential revision of the manuscript as outlined below:
   In the discussion the authors mention that ‘we included patients within the first 24 hours of mechanical ventilation’ however there is nothing in the methods to suggest only patients in the first 24 hours of mechanical ventilation were enrolled. Consequently, I am assuming that patients might have been on any day of their ICU stay when they were included in the study - this should be made clear.

Response: Thank the reviewer for addressing this important point. In fact, as described in the methods section we included patients that were in the ICU for at least 24 hours independently of the ventilatory support used and not within 24 hours of mechanical ventilation as mentioned in the discussion. We made the corrections of this information in this part of the discussion.

2- Further, it would be beneficial to provide an indication of how many patients were on day 1 day 2, day 3 etc of their ICU stay, otherwise this discussion point (lines 315 – 316) is not valuable as it is not possible to determine if, and how many, day 1 patients were included.

Response: Thank you for your suggestion, we do have information on the number of mechanically ventilated patients in the first 72 hours of ICU admission. And indeed only 27.2% [43/158] of mechanically ventilated patients were included in the first 72 hours of ICU admission and would in fact not be responsible for this difference. We agree with this point, and we made the necessary changes in this part of the discussion (Lines 288-308):

“Our results, in the mechanically ventilated patients, are in agreement with the results reported in a recent study performed in 11 ICUs located in southern Brazil ^(17)^. Nevertheless, the prevalence of mobilization in mechanically ventilated patients found in our study was higher than the prevalence between 32 to 45% reported by other authors ^(14-17)^. We believe that the high prevalence of mobilization in mechanically ventilated patients found in our study may be explained, at least in part, by the fact that, in Brazil, physiotherapists are part of the multidisciplinary ICU team assisting critically ill patients throughout the ICU stay.

Another important aspect, which differs from most international ICUs, is that Brazilian physiotherapists are part of the ICU team and are responsible for both the respiratory and mobilization therapy of critically ill patients. As a result, in what concerns patients that already have respiratory therapy prescription, such as mechanically ventilated patients, the physiotherapist has an independent practice to decide when to start mobilization, which may explain a high prevalence of mobilization in these patients. In most ICU patients, the usual decision to initiate mobilization in most Brazilian ICUs is related to the physician evaluation after patient ICU admission, in which case the decision to start happens earlier than physiotherapy evaluation. All the mobilization events reported in our study were led by physiotherapists; similar results were described by Fontela and colleagues ^(17)^.”

We have also included in the results information regarding the median ICU length of stay to the study day, and the number of patients included in the first 72 hours of ICU admission (lines 199-201):

“The median (IQR) length of ICU stay to the study day was 6 (3-13) days for all included patients. A total of 117 (32.6%) patients were included within 72 hours of ICU admission.”

We do have information regarding the barriers to achieve a higher activity level in the mechanically ventilated patients. We found a total of 202 barriers reported for these patients. The most frequent barrier reported was due to hemodynamics in patient [34/202; 16.8%], followed by the absence of early mobilization protocol [29/202; 14%] and excessive sedation [25/202; 12.3%].

Mechanically ventilated patients receiving passive exercise had a total of 190 reported barriers to achieve a higher level of activity. The most frequently reported barriers for these patients were the absence of early mobilization protocol [27/190; 14%] and access to specialized equipment [27/190; 14%], followed by patient’s hemodynamics [26/190; 13.7%] and excessive sedation [22/190; 11.5%]. These reported barriers may explain the higher incidence of passive exercise in mechanically ventilated patients when compared to the USA prevalence study.

We included this information in the results section (lines: 225-233).

An important difference between our study and the USA study that may be related to the difference of passive mobilization prevalence is the prevalence of physiotherapist involvement in mobilization therapies. In our study, 84.6% of mobilizations were performed by a physiotherapist, while in the USA study only 20% of physiotherapy involvement is found. In Brazil, the physiotherapist is part of the ICU team and is responsible for mobilizing patients, which may facilitate a higher number of patients being mobilized. This sentence was rewritten as follows (Lines 334-345):

“Passive exercises where the most frequent type of exercise performed in mechanically ventilated patients in our study. The prevalence of passive exercises in mechanically ventilated patients reported in the USA prevalence study was 62%, while our prevalence was 82.3% ^(16)^. This discrepancy may be related to the fact that the involvement of physiotherapist on patient’s mobilization included in the USA study was lower than our study, with only 20% involvement of physiotherapist compared to 84.6% respectively. This may be explained by the fact that physiotherapists in Brazil are part of the ICU team and have physiotherapy prescription for almost all mechanically ventilated patients. The barriers to achieve a higher activity level in mechanically ventilated patients reported in our study may also be responsible for a higher prevalence of passive exercises reported in this population.”

3- Assuming that my understanding above (that you included patients on any day of their ICU stay) is correct, I do not believe you can use the term ‘early mobilization’ throughout the manuscript as you have not measured ‘early’ mobilization, only ‘any’ mobilization in the ICU.

Response: We agree with the reviewer and his definition. We have removed the term "early" from the entire revised manuscript version.

We had initially used this term because previously point prevalence studies performed have also used this term using the same inclusion methodology as our study, including patients at any time during the ICU stay as described below:

- Fontela PC, Lisboa TC, Forgiarini-Júnior LA, Friedman G. Early mobilization practices of mechanically ventilated patients: a 1-day point prevalence study in southern Brazil. Clinics (Sao Paulo) 2018; 73:e241.
- Nydahl P, Ruhl AP, Bartoszek G, Dubb R, Filipovic S, Flohr HJet al. Early Mobilization of Mechanically Ventilated Patients: A 1-Day Point- Prevalence Study in Germany. Crit Care Med 2013; 42(5): 1178-86.

4- Lines 164 – 165: why was mobilization performed only by a physiotherapist, rather than by any member of the inter-disciplinary team, recorded as occurring? This is particularly important in the context that you discuss the importance of the inter-disciplinary team in your discussion, and at the very least needs to be included as a study limitation. This also impacts on your discussion (lines 305 – 306)

Response: Thank the reviewer for addressing this point. In fact, mobilization events were defined as any mobilization performed independently of which inter-disciplinary team performed it. During data collection this was not only restricted to physiotherapist mobilization. We have made the correction to this sentence in the manuscript in line 144 (methods section), as well in discussion.

5- Your findings indicate that half of the mobilisation, and almost all of the mobilisation in the mechanically ventilated patients, was limited to passive exercises – this is an important finding and needs to be conveyed in the abstract, results and conclusion, as well as discussed more fully. The result is that although there was a high level of mobilisation, there was almost no active mobilisation in the mechanically ventilated patients.

Response: We agree with the reviewer. We added this information in the abstract, as well as in the results and conclusion as suggested by the reviewer. In the discussion part we have also addressed this issue as suggested in Lines 334-345:

“Passive exercises were the most frequently performed type of exercise in mechanically ventilated patients in our study. The prevalence of passive exercises in mechanically ventilated patients reported in the USA prevalence study was 62%, while our prevalence was 82.3% ^(16)^. This discrepancy may be related to the fact that involvement of a physiotherapist in patient’s mobilization included in the USA study was lower than our study, with only 20% involvement of physiotherapist compared to 84.6% respectively. This may be explained by the fact that physiotherapists in Brazil are part of the ICU team and there is physiotherapy prescription for almost all mechanically ventilated patients. Barriers to achieving a higher activity level in mechanically ventilated patients reported in our study may also be responsible for a higher prevalence of passive exercises reported in this population.”

6- Page 6 – please clarify that individual patients did not need to provide consent

Response: The ethics committee requested informed consent for all patients included in the study. We added this information in the manuscript in lines 112-113 as follows:

“Informed consent was obtained for all patients as requested by the ethics committee.”

7- Line 188 – you indicate that 26 of 100 invited ICUs participated, but do you have data on how many ICUs exist in Brazil? If so, this should be included. Also, did the included ICUs represent those invited and the entire Brazilian cohort of ICUs in terms of type and size? This also needs to be incorporated into the relevant section of your discussion (lines 335 – 337).

Response: We thank the reviewer for the suggestion. Unfortunately, we do not have the exact number of ICUs in Brazil. Recently, the Federal Physician Council in Brazil reported a total of 45,000 ICU beds, but these are present in only 10% of Brazilian counties cities. They reported 49% of public ICUs, and in our study 51% of the ICUs included were public ICUs. Most Brazilian ICUs are located in southern Brazil (60%), and the rest in the north and midwest Brazil. In our study 65.3% of ICUs were from the southeast and south of Brazil, 26.8% from the northeast and northern region, and 7.7% from the midwest. The ICUs included represent those invited in terms of type and size. This information was incorporated into the relevant section of our discussion as suggested by the reviewer. (Lines 367-374).

8- Page 9 and elsewhere – the patient to nurse ratio is extremely high for many ICUs internationally, and the average SOFA score is very low; linked to this is that only 44% of patients were mechanically ventilated and a further 12% non-invasively ventilated, leaving 44% not requiring ventilation support at all. This combination of data raises questions about whether the included units were all ICUs or whether some were more representative of what might be referred to as a High Dependency Unit and how this context compares internationally; discussion of this should be included.

Response: Thank the reviewer for the comments.

Regarding the patient to nurse ratio, in Brazil the ratio is actually higher than in other countries according to recommendation of the Brazilian Federal Nursing Council. This recommendation is due to the fact that in Brazil ICUs have also nursing assistants, who are also responsible for direct patient care while nurses have a more managerial position for these nursing assistants. Based on the Brazilian Federal Nursing Council recommendation, the patient to nursing assistant ratio should be 1:2, and the patient to nurse ratio should be 1:6, according to our study findings. Similar results can be found in the study published by Fontela and colleagues (Fontela PC, Lisboa TC, Forgiarini-Júnior LA, Friedman G. Early mobilization practices of mechanically ventilated patients: a 1-day point prevalence study in southern Brazil. Clinics (Sao Paulo) 2018; 73:e241). This information was included in lines 311-318.

The SOFA score in fact is very low. This is probably due to the fact that most patients did not require invasive mechanical ventilation and had low hepatic dysfunction at the study day. As SOFA score was measured for the study day in order to correlate with the mobilization practice, these facts may have influenced the low SOFA observed in our study population.

All units included were ICUs. The low SOFA score and low prevalence of patients under mechanical ventilation may be explained by the fact that in our study all ICUs patients that met the inclusion criteria were included in the study and may have been included during an improvement in their clinical setting during ICU stay. The same inclusion methodology as previously prevalence studies performed.

This discussion was included as follows (lines 346-352):

“In our study the average SOFA score was very low. This finding is probably due to the fact that most patients did not require invasive mechanical ventilation at the study day, which influences the SOFA score. The SOFA score was measured for the study day in order to correlate with the mobilization practice. Another aspect to consider is that patients were included on any day during ICU stay, with the majority of patients on the sixth day of ICU admission, and they may have been included during an improvement in their clinical setting.”

9- Line 206 – does 358 represent all patients who were in the participating ICUs on the study day, or were some excluded and if so how many and why? This information needs to be included in figure 1.

Response: The 358 represent all patients who were in the participating ICUs on the study day. No patient was excluded.

10- Lines 235 – 237: it would be useful to understand the usual decision making in the practice setting, i.e. do physios normally have independent practice or is it a policy requirement that physicians need to make the decision about who and when to mobilise? Linked with this is detail regarding the early mobility protocols that you refer to – what was normally included in these?

Response: The usual decision making to initiate mobilization in most Brazilian ICUs is related to physician evaluation after patient’s ICU admission, in which case the decision to start occurs earlier than a physiotherapy evaluation. Brazilian physiotherapists are part of the ICU team and are responsible for both respiratory and mobilization therapy of critically ill patients. Consequently, in what concerns patients that already have respiratory therapy prescription, the physiotherapist has an independent practice to decide when to start mobilization. This information was included in the discussion in lines 297-306.

The mobility protocols usually start with patient evaluation and clinical criteria to start mobilization, as hemodynamic and respiratory reserve and without any contraindications. Based on this evaluation, patient may be included in one of the 4 phases of mobilization as previously published and recommended ^(11,12,24)^. This information was included in the discussion section describing the characteristics of participating center in lines 329-333.

11- Line 251: can you give more information about the contra-indications and who decided that these were contra-indications?

Response: Contra indications were considered as respiratory, cardiovascular, neurological or other considerations as described in the study published by Hodgson and colleagues ^(21)^. These contra indications were decided by the researchers responsible to collect the data on the study day based on the Hodgson and colleagues previously published study criteria. This information was included in the manuscript in lines 149-152.

Hodgson CL, Stiller K, Needham DM, Tipping CJ, Harrold M, Baldwin CE et al. Expert consensus and recommendations on safety criteria for active mobilization of mechanically ventilated critically ill adults. Critical Care 2014; 18: 658.

12- Lines 251 – 252: it’s not clear to me while the absence of a mobility protocol is a barrier – please clarify.

Response: As described in the study published by Dubb R and colleagues, the absence of a mobility protocol is considered a structural barrier for early mobilization. Without a mobility protocol the ICU team will not be able to identify the safety criteria to start mobilization and nor a standardized protocol to be followed by all members of the team. Previously published studies ^(10-13)^ have also shown that the presence of an early mobility protocol, when compared to usual care, improves hospital length of stay, mechanical ventilation duration, delirium, and patients get out of bed earlier. This information was also included in the discussion in lines 322-328.

Dubb R, Nydahl P, Hermes C, Shwabbauer N, Toonstra A, Parker AM et al. Barriers and strategies for early mobilization of patients in intensive care units. Ann Am Thorac Soc 2016; 13(5): 724-730.

13- Tables 3 and 4 – analysis:
    - I would expect number of patients per physiotherapist and number of physiotherapists per 6-hour shift to potentially be highly correlated; was multi-collinearity checked for? Similarly, can you confirm that multi-collinearity between type of hospital and SOFA score was checked for?

Response: Multi-collinearity was checked for all variables. As follows, all VIFs (variance inflation factors) are lower than 5. We included this information in the manuscript and in the supporting information file. We have included this information in the Statistical Analysis (line 164) and supporting information file in S1 Table.

|  | VIF Df | VIF^(1/(2*Df) |
| --- | --- | --- |
| Table 3 |  |  |
| SOFA | 2.427044  1 | 1.557897 |
| factor(vent_support) | 1.825397  2 | 1.162356 |
| factor(vasoactive) | 1.627499  1 | 1.275735 |
| number_physio | 2.438260  1 | 1.561493 |
| number_pat_physio | 2.266605  1 | 1.505525 |
| factor(protocol_insti) | 2.064531  1 | 1.436848 |
| factor(type_hospital) | 5.343080  2 | 1.520365 |
| Table 4 |  |  |
| SOFA | 1.246340  1 | 1.116396 |
| factor(vent_support) | 1.106374  2 | 1.025594 |
| factor(vasoactive) | 1.193238  1 | 1.092354 |
| number_physio | 1.638603  1 | 1.280079 |
| number_pat_physio | 1.413337  1 | 1.188839 |
| factor(protocol_insti) | 1.515191  1 | 1.230931 |
| factor(type_hospital) | 1.701053  2 | 1.142035 |

- It is not clear to me why you have entered ‘invasive mechanical ventilation’ and ‘noninvasive ventilation’ as 2 separate factors in the regression, rather than ‘type of ventilation’ being the factor with 3 possible response categories (invasive, non-invasive, no ventilation support – this latter category should be the reference category)

Response: We apologize for being unclear. We did use Type of ventilation support as being the factor with 3 possible response categories as you mentioned. We included this information in the result table as follows:

| **Variables** | **OR** | **95% CI** | **p value** |
| --- | --- | --- | --- |
| SOFA score | 0.72 | 0.60 - 0.86 | < 0.001 |
| Ventilatory support |  |  |  |
| No support | 1.00 | (Reference) | --- |
| Noninvasive ventilation | 0.31 | 0.10 - 0.97 | 0.04 |
| Invasive mechanical ventilation | 0.13 | 0.04 – 0.41 | < 0.001 |
| Use of vasoactive drugs  Type of hospital  Public  Private  University | 1.18  1.00  1.63  2.81 | 0.34 - 4.03  (Reference)  0.54 – 4.96  0.79 – 9.94 | 0.79  ---  0.38  0.10 |
| Number of physiotherapists per 6-hour shift | 1.17 | 0.90 - 1.52 | 0.24 |
| Number of patients per physiotherapist | 1.30 | 1.07 – 1.59 | < 0.001 |
| Institutional early mobility protocol | 5.80 | 1.33 - 25.30 | 0.02 |

- Type of hospital – given you have OR for 3 different categories it is not clear to me what the reference category was – can you clarify?

Response: We apologize for being unclear, our reference category for type of hospital was Public, and a correction to this information was made on the result table as well. We included this information in the result table as follows:

Table 3

| **Characteristics** | **OR** | **95% CI** | **p value** |
| --- | --- | --- | --- |
| SOFA score | 0.95 | 0.84 - 1.07 | 0.39 |
| Ventilatory support |  |  |  |
| No support | 1.00 | (Reference) | --- |
| Invasive mechanical ventilation | 1.46 | 0.45 - 4.73 | 0.52 |
| Noninvasive ventilation | 1.37 | 0.55 - 3.42 | 0.49 |
| Use of vasoactive drugs | 0.89 | 0.37 - 2.14 | 0.80 |
| Type of hospital  Public  Private  University | 1.00  1.04  0.38 | (Reference)  0.27 - 3.97  0.12 - 1.16 | ---  0.95  0.09 |
| Number of physiotherapists per 6-hour shift | 0.82 | 0.59 - 1.12 | 0.21 |
| Number of patients per physiotherapist | 1.09 | 0.93 - 1.27 | 0.29 |
| Institutional early mobility protocol | 3.19 | 1.23 - 8.22 | 0.01 |

Table 4

| **Variables** | **OR** | **95% CI** | **p value** |
| --- | --- | --- | --- |
| SOFA score | 0.72 | 0.60 - 0.86 | < 0.001 |
| Ventilatory support |  |  |  |
| No support | 1.00 | (Reference) | --- |
| Noninvasive ventilation | 0.31 | 0.10 - 0.97 | 0.04 |
| Invasive mechanical ventilation | 0.13 | 0.04 – 0.41 | < 0.001 |
| Use of vasoactive drugs  Type of hospital  Public  Private  University | 1.18  1.00  1.63  2.81 | 0.34 - 4.03  (Reference)  0.54 – 4.96  0.79 – 9.94 | 0.79  ---  0.38  0.10 |
| Number of physiotherapists per 6-hour shift | 1.17 | 0.90 - 1.52 | 0.24 |
| Number of patients per physiotherapist | 1.30 | 1.07 – 1.59 | < 0.001 |
| Institutional early mobility protocol | 5.80 | 1.33 - 25.30 | 0.02 |

14- Lines 285 – 287: did you look at how long the safety events lasted, and whether any intervention was required to resolve the safety issue?

Response: Thank you for your suggestion, but unfortunately we do not have information regarding how long the safety events lasted or whether any intervention was required to resolved it.

15- Lines 324 – 327 – I am a little uncertain how the number of students available might affect mobilisation rates when you only considered mobilisation performed by a physiotherapist – please clarify.

Response: Thank the reviewers for the considerations. In Brazil, university hospital usually have physiotherapy students and they support the clinical physiotherapist during mobilization therapy. As for out of bed exercises, patients may need more than one person to perform mobilization. Therefore, having a student present may help achieve this goal, especially when there is unavailable staffing to perform it. Although, after revising our statistical analysis we found no association of university hospitals with out of bed exercise and for this reason we have removed this in our discussion section.

16- Minor comments:
    - Please write ‘colleagues’ out in full rather than ‘cols’

Response: We changed this term “cols” to “colleagues” in the manuscript as suggested by the reviewer.

**Reviewer #2:**

Thank you very much for your comments and suggestions.

This is a very promising manuscript presenting mobilization data of a 1-day prevalence study in Brazil. Before accepting the manuscript, however, several major points should be clarified in my point of view.

1- This prevalence study is not investigating early mobility or early mobilization because it investigates all ICU patients regardless of the length of the ICU stay. Therefore, the manuscript should be rephrased accordingly and “early” should be omitted throughout. Furthermore, the term mobilization or mobilization therapy should be used instead of mobility, because the patients are mobilized mainly.

Response: We agree with the reviewers and its definition. We have removed the term "early" from the entire revised manuscript version.

We had initially used this term because previously point prevalence studies performed also used this term using the same inclusion methodology as our study, including patients at any time during the ICU stay as described below:

- Fontela PC, Lisboa TC, Forgiarini-Júnior LA, Friedman G. Early mobilization practices of mechanically ventilated patients: a 1-day point prevalence study in southern Brazil. Clinics (Sao Paulo) 2018; 73:e241.
- Nydahl P, Ruhl AP, Bartoszek G, Dubb R, Filipovic S, Flohr HJet al. Early Mobilization of Mechanically Ventilated Patients: A 1-Day Point- Prevalence Study in Germany. Crit Care Med 2013; 42(5): 1178-86.

The term mobility was changed to mobilization or mobilization therapy as suggested by the reviewers.

2- That said, it would be interesting to see early mobilization data. Do the authors have the ICU day of the patients so that they can provide data on early mobilization (e.g. within 72 hours of ICU admission)?

Response: Thank you for your suggestion. We do have data regarding how many patients were included within 72 hours of ICU admission: a total of 117 (32.6%) out of the 358 patients were included within 72 hours of ICU admission. We included this data in the result section (Lines 199-201):

“The median (IQR) length of ICU stay to the study day was 6 (3-13) days for all included patients. A total of 117 (32.6%) patients were included within 72 hours of ICU admission.”

3- You define your “mobilization therapy” by the ICU mobility scale. The ICU mobility scale, however, is a mobilization therapy strategy only incorporating active mobilization forms, while you use passive, active-assisted and active forms. Please change your definition accordingly. Since you do not use the IMS in the presentation of the data, you could omit it? Otherwise, describe your changed definition.

Response: Thank you for your suggestion and we agree with it. We have made the necessary changes in the manuscript and did not use the ICU Mobility Scale. (Lines 146-149).

4- Furthermore, I have strong objections of your definition of in- and out-of bed exercises. Sitting (passively in a chair) and sitting at the edge (IMS 3) of bed is a typical out of bed exercise while passive mobilization, sitting in the bed and exercises like bedergometry are typical in-bed exercises (ICU Mobility Scale = 1). Please provide references which have used the definitions you used or change your presentation of the data accordingly.

Response: Thank the reviewers for the comments. We have changed our presentation of the data accordingly. (Table 2 – page 11)

5- How many ICUs are there in Brazil (approximately)?

Response: Thank the reviewers for the suggestion. Unfortunately, we do not have the exact number of ICUs in Brazil. Recently, the Federal Physician Council in Brazil reported a total of 45,000 ICUs beds; however, these are present in only 10% of Brazilian counties cities. They reported 49% of public ICUs, and in our study 51% of the ICUs included were public ICUs. Most Brazilian ICUs are located in southern Brazil (60%), and the rest in the north and midwest. In our study, 65.3% of ICUs were from the southeast and south of Brazil, 26.8% from the northeast and north region, and 7.7% from the midwest region. The ICUs included represent those invited in terms of type and size. This information was incorporated into the relevant section of our discussion (lines 367-374).

6- Please provide the study protocol as supplement.

Response: We have included the study survey as well as the study protocol as supplement as suggested by the reviewers.

7- Please provide the appropriate checklist from the https://www.equator-network.org/ as supplement

Response: The appropriate checklist has been provided as supplement as suggested by the reviewers.

8- You have not provided your dataset as required by PLOS One.

Response: We have made the necessary changes as suggested by the reviewers and provided the dataset as required by PLOS One.

9- Because of these major points the result section and discussion section will have to go substantial changes and will have to be assessed then. For the discussion section, I think the comparison with the other 1-day prevalence studies and differences to their results should be discussed in more detail, since the methods were not identical. Consequently, the prevalence numbers cannot be compared without highlighting the differences.

Response: We have made the necessary and suggested changes to the major points raised by the reviewers and also made changes regarding the differences and comparison with the other 1-day point prevalence studies.

Minor points:
Background:
The authors should reconsider references 5-9 for the statement line 109/110. There is a recent metanalysis for example available (https://doi.org/10.1371/journal.pone.0223185) addressing this point

Response: Thank the reviewers for the suggestion. We have included the recent meta-analysis suggested. We included it as reference 9.

10- The references 10-13 should be reconsidered as well. Please be accurate with mobilization RCTs which improved independent physical status at hospital discharge, which ones improved ventilation days, etc.

Response: We have made the necessary changes regarding the statements provided. And included new RCTs references to address this point also (Lines 86-90).

11- Please change “and cols.” to “et al.” through the manuscript

Response: We have changed “and cols, to “colleagues” throughout the manuscript as suggested by the other reviewer as well.

Methods

12- Please provide the date of the prevalence study.

Response: The prevalence study was performed on June 29^th^, 2017. We included this information in the method section.

13- Please omit reference 18. Redcap does not need 2 references.

Response: We have omitted reference 18 as suggested by the reviewers.

14- Please explain what 6-hour shift means. Does that mean that in Brazil you have physical therapy during 24 hours and you have 4 shifts of 6 hours? Is the ratio unvaried over the day?

Response: A 6-hour shift means that physical therapy usually has a 6-hour shift during at least 18 hours as recommended by our council, and for this reason ICUs usually have at least 3 shifts of 6 hours. Some ICUs have a 12-hour shift during the day and 6 hour-shift at night. The number of patients per physiotherapist takes into account a 6-hour shift with a new distribution of patients on the next 6-hour shift.

15- P. 7 line 160/161: “related to the study day”. Please rephrase since ICU admission diagnosis was not.

Response: We rephrased the sentence as suggested by the reviewers (lines 139-141):

“The following patients’ variables were related to the study day: SOFA, supportive therapy, type of ventilatory support, sedation practices and mobilization activities.”

16- You use SOFA and need of vasopressors as confounders. Since vasopressors are in the SOFA score, please check for collinearity and provide information.

Response: Multi-collinearity was checked for all variables. As follows, all VIFs (variance inflation factors) are lower than 5. We included this information in the manuscript and in the supporting information file. We have included this information in the Statistical Analysis (line 164) and supporting information file in S1 Table.

|  | VIF Df | VIF^(1/(2*Df) |
| --- | --- | --- |
| Table 3 |  |  |
| SOFA | 2.427044  1 | 1.557897 |
| factor(vent_support) | 1.825397  2 | 1.162356 |
| factor(vasoactive) | 1.627499  1 | 1.275735 |
| number_physio | 2.438260  1 | 1.561493 |
| number_pat_physio | 2.266605  1 | 1.505525 |
| factor(protocol_insti) | 2.064531  1 | 1.436848 |
| factor(type_hospital) | 5.343080  2 | 1.520365 |
| Table 4 |  |  |
| SOFA | 1.246340  1 | 1.116396 |
| factor(vent_support) | 1.106374  2 | 1.025594 |
| factor(vasoactive) | 1.193238  1 | 1.092354 |
| number_physio | 1.638603  1 | 1.280079 |
| number_pat_physio | 1.413337  1 | 1.188839 |
| factor(protocol_insti) | 1.515191  1 | 1.230931 |
| factor(type_hospital) | 1.701053  2 | 1.142035 |

17- Provide a definition of “sedation” (used in table 1) in the methods section

Response: The definition of “sedation” was included in the methods section as suggested by the reviewers. The “sedation” used in table 1 refers to the number of included patients that were using any type of sedation on the study day.

Discussion

18- P. 14 line 295: change “during the ICU stay” to “that day” or “on XX / XX / 20XX)

Response: We have changed in the discussion as suggested by the reviewers. Now in line 285.

19- Line 313-314: In that case I believe that your very good physical therapy staffing might have an influence, since passive mobilization is done by them. I would consider bringing up that argument there

Response: Thank the reviewers for the suggestion we have made the following argument as also recommended by the other reviewer (Lines 334-345):

“Passive exercises where the most frequent type of exercise performed in mechanically ventilated patients in our study. The prevalence of passive exercises in mechanically ventilated patients reported in the USA prevalence study was 62%, while our prevalence was 82.3% ^(16)^. This discrepancy may be related to the fact that the involvement of physiotherapist on patient’s mobilization included in the USA study was lower than our study, with only 20% involvement of physiotherapist compared to 84.6% respectively. This may be explained by the fact that physiotherapists in Brazil are part of the ICU team and have physiotherapy prescription for almost all mechanically ventilated patients. The barriers to achieving a higher activity level in mechanically ventilated patients reported in our study may also be responsible for a higher prevalence of passive exercises reported in this population.”

20- Please omit reference 22.

Response: We have omitted the reference 22 from the manuscript as suggested by the reviewers.

21- Line 324: Please change to “University hospitals in Brazil…” if this statement is really true, that students support mobilization therapy at your ICUs. This is not a general truth.

Response: Thank the reviewers for the considerations. In Brazil, university hospital usually have physiotherapy students and they support the clinical physiotherapist during mobilization therapy. As for out of bed exercises, patients may need more than one person to perform mobilization. Therefore, having a student present may help achieve this goal, especially when there is unavailable staffing to perform it. Although, after revising our statistical analysis we found no association of university hospitals with out of bed exercise and for this reason we have removed this in our discussion section.

22- Limitations: The generalizability depends on the total number of ICUs in Brazil. See question above.

We have added information regarding the total number of ICUs in Brazil and how the included ICUs could represent them in our study as described in a question answered above to the reviewers. We have also included this information in the manuscript. (Lines 367-374)

23- Please explain the blinding to the team, since the patients had to give their consent (how was that not seen by the team). Or was the consent waived by the IRB (if so, please change the statement in the methods section accordingly)

Response: The team responsible for the patient care was not aware of the study and the data that was collected from the nursing or physiotherapy notes as well as from the daily observation chart. The data was collected by the research team, which was not responsible for the patient care. Patients gave their consent to the research team after the 24-hour follow up in order to not influence the results (posteriori informed consent as there was no intervention specifically defined for the study).
