# Supplementary material for: Mobilization practices in the ICU: A nationwide 1-day point- prevalence study in Brazil
Source: PLoS One. 2020 Apr 2;15(4):e0230971. doi: 10.1371/journal.pone.0230971 (PMC7117707; doi:10.1371/journal.pone.0230971)
Supplement: Attachment — Submitted filename: Response to Reviewers 2.docx [file pone.0230971.s003.docx]

São Paulo, March 9^th^, 2020.

Response to the Editor’s and the Reviewers’ comments

**PONE-D-19-29730
Mobilization practices in the ICU: A nationwide 1-day point- prevalence study in Brazil**

Dear Martina Crivellari,

Academic Editor of PLOS ONE

We would like to express our gratitude for giving us the opportunity to revise our manuscript. We believe the reviewers made insightful suggestions that have greatly helped address critical unresolved issues to improve the manuscript.

All the changes in the paper are highlighted in yellow in the revised version to indicate the revised portions of the manuscript. We made modifications in the manuscript according to the reviewers’ comments keeping it within the word and referenced limits imposed by the Journal. We have also revised the manuscript taking into consideration the request made by the Editorial Office. We expect our manuscript to be now suitable for publication in PLOS ONE.

I remain at your disposal for any clarification you might require.

Yours sincerely,

On behalf of all the authors,

Karina T. Timenetsky

**Reviewer #1:**

Thank you very much for your additional comments and suggestions.

1- Thank you for submitting a much improved version of this manuscript. Despite the improvements I do have a small number of remaining queries and suggestions as follows:
   Line 122: why were pregnant patients excluded?

Response: We excluded pregnant patients due to the possible mobilization limitation that these patients may present related to pregnancy itself.

2- Lines 149 – 151: please add in notation of who determined that a contra-indication was present

Response: Thank you for your suggestion. We added a notation of who determined that a contra indication was present as follows:

“Contra indications for mobilization were considered as respiratory, cardiovascular, neurological or other considerations as described in the study published by Hodgson and colleagues ^(21)^. Contra indication was considered present after the health care and research team reached consensus on this topic. “ (Lines 157 – 158)

3- Line 155: this information belongs in the ‘participant information’ section

Response: Thank you. We have included this information in the “participant information” as the reviewer recommended.

4- Line 190: given you have nursing assistants as well in your environment, the ratio for them should also be reported as the patient to nurse ratio is meaningless in isolation

Response: Thank the reviewer for the comments. We have added this information in the result section. Lines:_195-196._____

“The median (IQR) patient to nursing assistant ratio was 2 (2.0-3.0).” This ratio is in accordance to the Nursing Federal Council recommendation.

5- Line 193: thank you for clarifying that individual patient consent was required; it is highly unusual to have 100% of ICU patients consent to a study (I do not believe I have ever seen it before) so it needs to be made clear in figure 1 that 358 was the total number of patients in study ICUs on the relevant day, and that 358 patients were recruited. In the text I suggest you outline the process used to obtain consent.

Response: Thank the reviewer for the comments. In fact, 100% for informed consent is very high, but due to the nature of the study design, where there was no intervention made, patients and families usually have no restrictions in signing the informed consent.

The process used to obtain consent involved approaching the family member or patient when they met the study inclusion criteria, then explain the objective of the study, and the possible uses of the information obtained from it. The person responsible for the study in each center was responsible for obtaining consent. This information was included in lines 112-116. We have also made changes in Figure 1 as suggested.

6- Line 199: this is an extremely long ICU LOS, particularly in the context of the very low SOFA – there needs to be further description of the context and usual practices to explain why patients that predominantly do not require invasive mechanical ventilation spend about a week in ICU as this is very different to most international practice and possibly accounts for why your mobilisation rates are high.

Response: Our SOFA score represents the study day SOFA and not the ICU admission SOFA and as the majority of patients were included after 72 hours of ICU admission, patients may have improved during ICU stay. Another important point is that most hospitals do not have step down units, and patients may stay in the ICU longer in order to be clinically stable before receiving ward discharge. Similar ICU LOS, with a median (IQR) of 7 (3-7) days was also described by Fontela and colleagues. In Brazil, as reported by the Brazilian Intensive Medicine Association, the mean ICU LOS in 2017 was 16 days.

7- Lines 292 – 296: although the role of the physiotherapist might contribute to the mobilisation level you have reported, I suggest that the very low severity of illness and the relatively long ICU length of stay reported in your cohort might contribute as much, if not more, to the opportunity for mobilisation. The influence of these characteristics is discussed much later in the discussion (lines 346 – 352) and needs to be moved earlier to integrate with this discussion.

Response: We agree with the reviewer and have made the necessary changes moving the discussion of SOFA and length of stay earlier in the discussion as suggested. Lines: 298-311.

8- Lines 297 – 299: I actually disagree with this statement – there are many areas of the world (including UK, some parts of Europe, Australia & New Zealand, possibly others) where the physiotherapists are part of the ICU team responsible for both respiratory and mobilisation therapy; I tend to think you are making your comparison with the USA which is notable for different therapists undertaking different elements of care and not always being part of the ICU team but this does not reflect much of international practice.

Response: We agree with the reviewer and have made changes to this statement in the discussion. Lines: 312-313.

9- Lines 340 – 342 – repetitive of previous page – suggest deleting

Response: We agree with the reviewer and excluded this information.

10- Lines 368 – 373: this information repeats what has been provided earlier in the
    Limitations: the lack of information about the severity and duration of adverse events should be added as a limitation

Response: We have removed this information and included the lack of information about the severity and duration of adverse events in the limitation section.

11- Typographical comments:
    a) Lines 186 – 187 and elsewhere – ‘respectively’ is often better located after the relevant data or at the end of a sentence, for example ‘… delirium protocols were reported in 50% (13/26) and 38.5% (10/26), respectively, of the ICUs’

Response: Thank the reviewer, we have made the proper corrections in the manuscript.
b) Line 334 – should be ‘were’ rather than ‘where’

Response: Thank the reviewer, we have made the proper corrections in the manuscript.
c) Line 360 – delete ‘P’ after ‘Nydahl’

Response: Thank the reviewer, we have made the proper corrections in the manuscript.

**Reviewer #2:**

Thank you very much for your additional comments and suggestions.

1- The format of the references must be corrected. PLOS One does not use superscript references.

Response: We have made the proper correction related to the format of the references in the manuscript. We have changed to square brackets as recommended.

2- I would be very interested to see data on early mobilization (i.e. within 72 hours of ICU admission). You state that 32.6% fall into this category. Please provide information in the appendix similar to table 3 and 4 in the appendix in this cohort

Response: Thank the reviewer for the suggestion. We have provided information in the appendix similar to table 3 and 4 in patients within 72 hours as suggested by the reviewer. This information is described in S2 Table and S3 Table.

3- The main finding of the study – having an early mobility protocol – is part of the first paragraph of the discussion, but not adequately discussed in the discussion section and not mentioned as a conclusion.

Response: Thank the reviewer for addressing this important point. We have discussed this point in the discussion section and included in the conclusion as suggested as well.

4- One concern still exists and has to be clarified before that manuscript can be accepted. Both reviewers asked for the numbers of total ICUs so that it can be adequately assessed what the external validity of your findings is. Providing the total number of ICU beds is not satisfactory for that! Accounting the median size of ICUs in your study and use that to calculate the ICU numbers based on the presented number of 45.000 beds, resulting in 2500 ICUs. That would mean that you present data of 1% of ICUs in Brazil. Please provide the accurate number or this estimation in the manuscript to provide the reader with the adequate context and limitation.

Response: We included the total number of ICUs in the discussion section as suggested by the reviewers (total number of 1291 ICUs). Lines: 375

5- Just to clarify, you answered that there was no patient excluded. So you had a 100% success rate in getting your deferred consent. Is that correct?
   Response: In fact, 100% for informed consent is very high, but due to the nature of the study design, where there was no intervention made, patients and families usually have no restrictions in signing the informed consent.
6- Abstract
   - Cut the listening of the mobilization levels with %

Response: We have made the necessary changes as suggested by the reviewers.

7- - Present the main finding – besides frequency of mobilization (especially passive) as you did, present the findings what influences mobilization (early mobility protocol). This is your message! Revise Conclusion accordingly

Response: Thank the reviewers for the suggestion. We have made the necessary change as suggested.

8- Introduction
   - P. 4, line 86: Please add “, if applied early,” in the sentence, so it reads “it improves, if applied early, independent physical status at hospital discharge…”

Response: We added “if applied early” in the sentence as suggested by the reviewers. (Line: 85-86)

9- Results
   - p 8 study population: please shorten the description with main points (e.g. sex and age), referring to table 1 without repetition of all numbers in the text.

Response: We have made the necessary changed as suggested by the reviewers. (lines 199-206)

- p.12 line 248. Please add a sentence that no collinearity was present.

Response: We have added a sentence as suggested by the reviewers.(Line 251)

- p.12/13 line 261-266: It is not necessary to repeat all factors if they are presented in the table. Feel free to state the significant one but do not repeat all please.

Response: We have made the necessary changed as suggested by the reviewers. (261-266)

10- Discussion
    - p. 14, line 286 please change “The vast majority of patients receiving mechanical ventilation included in our study received mobilization, but mostly no active mobilization was performed

288 in these patients.“ To “The vast majority of patients receiving mechanical ventilation included in our study received passive mobilization.”

Response: We have made the necessary changed as suggested by the reviewers. (line: 288-289)

- p.13, line 299-302: Sentence has to be revised for better understanding and English grammar

Response: We have revised the sentence as suggested by the reviewers.(Line: 315-319)

- p. 16 line 340-342: Repetition of p.13

Response: We have excluded this sentence in p.16 as suggested by the reviewers.

11- Table 2
    - Line 236: “According to” – the O is missing.

Response: Thank the reviewers for addressing this point. We have made the necessary correction as raised by the reviewers.
